# Skills for generalist and specialist nurses working in the prevention and control of infections in Brazil*

**DOI:** 10.1590/1518-8345.2620.3134

**Published:** 2019-04-29

**Authors:** Aline Massaroli, Jussara Gue Martini, José Luis Medina Moya, Milca Severino Pereira, Anaclara Ferreira Veiga Tipple, Eleine Maestri

**Affiliations:** 1 Universidade Federal da Fronteira Sul, Chapecó, SC, Brasil.; 2 Universidade Federal de Santa Catarina, Departamento de Enfermagem, Florianópolis, SC, Brasil.; 3 Universidade de Barcelona, Departamento de Pedagogia, Barcelona, Espanha.; 4 Pontifícia Universidade Católica de Goiás, Departamento de Enfermagem, Goiânia, GO, Brasil.; 5 Universidade Federal de Goiás, Faculdade de Enfermagem, Goiânia, GO, Brasil.

**Keywords:** Nursing, Infection Control, Professional Competence, Higher Education, Nursing Education, Prevention and Control, Enfermagem, Controle de Infecções, Competência Profissional, Educação Superior, Educação em Enfermagem, Controle e Prevenção, Enfermería, Control de Infecciones, Competencia Profisional, Educación Superior, Educación en Enfermería, Control y Prevención

## Abstract

**Objective:**

to define the competencies for the prevention and control of healthcare-related infections that should be developed by the generalist nurse and the specialist nurse in infection control in Brazil.

**Method:**

the Delphi technique, developed in four rounds, was used. Thirty-one nurses and eight physicians participated in the study, with expertise in infection prevention and control. Data were collected using open-ended questionnaires, whose answers were treated using the content analysis technique. Structured instruments were used to evaluate the importance of each competency using a Likert scale. Data were analyzed and presented in a descriptive way, use of median and coefficient of variation.

**Results:**

the competences were organized in 4 core, 14 generic and 17 specific, with name and description of each competency.

**Conclusion:**

the definition of competencies for the prevention and control of healthcare-related infections is the first step to begin the rethinking of the teaching and learning process in the initial training of nurses. The data found in the present study may help to restructure education and support permanent education programs in health.

## Abstract (pt)

**Objetivo:**

definir as competências para a prevenção e o controle de infecções relacionadas à assistência à saúde que devem ser desenvolvidas pelo enfermeiro generalista e pelo enfermeiro especialista em controle de infecções no Brasil.

**Método:**

empregou-se a técnica Delphi, desenvolvida em quatro rodadas. Participaram do estudo 31 enfermeiros e oito médicos com *expertise* em prevenção e controle de infecções. Para a coleta de dados, utilizaram-se instrumentos com perguntas abertas cujas respostas foram tratadas por meio da técnica de análise de conteúdo e instrumentos estruturados para avaliar a importância de cada competência por meio de escala Likert, sendo os dados analisados e apresentados de forma descritiva, uso de mediana e de coeficiente de variação.

**Resultados:**

as competências foram organizadas em 04 centrais, 14 genéricas e 17 específicas, com nome e a descrição de cada competência.

**Conclusão:**

a definição das competências para a prevenção e o controle de Infecções Relacionadas à Assistência à Saúde é o primeiro passo para se iniciar o repensar acerca do processo de ensino e aprendizagem na formação inicial dos enfermeiros. Os dados encontrados auxiliam na reestruturação do ensino, além de fundamentar programas de educação permanente em saúde.

## Abstract (es)

**Objetivo:**

definir las competencias para la prevención y el control de infecciones relacionadas a la asistencia a la salud que deben ser desarrolladas por el enfermero generalista y por el enfermero especialista en controle de infecciones en Brasil.

**Método:**

se empleó la técnica Delphi, desarrollada en cuatro rodadas. Participaron del estudio 31 enfermeros y ocho médicos, con experiencia en prevención y control de infecciones. Para la recolección de datos se utilizaron instrumentos con preguntas abiertas, cuyas respuestas fueron tratadas por medio de la técnica de análisis de contenido, e instrumentos estructurados para evaluar la importancia de cada competencia por medio de escala Likert, siendo los dados analizados y presentados de forma descriptiva, uso de mediana y de coeficiente de variación.

**Resultados:**

las competencias fueron organizadas en 04 centrales, 14 genéricas y 17 específicas, con nombre y la descripción de cada competencia.

**Conclusión:**

la definición de las competencias para la prevención y el control de Infecciones Relacionadas a la Asistencia a la Salud es el primer paso para iniciarse el repensar acerca del proceso de enseñanza y aprendizaje en la formación inicial de los enfermeros. Los datos encontrados auxilian en la reestructuración de la enseñanza además de fundamentar programas de educación permanente en salud.

## Introduction

The teaching of prevention and control of healthcare-related infections (HCRI) has been pointed out by national^(^ ^1^ ^-^ ^4^ ^)^ and international studies^(^ ^5^ ^-^ ^9^ ^)^ as an area of great weaknesses related to the knowledge of health professionals on this topic, which reflects in the care practice, where there is great unpreparedness of the health team to employ the necessary measures for the prevention and control of HCRI. Also, the teaching of this area needs to be rethought and restructured aiming to establish professional competences must be learned from the beginning of the nurse’s professional training.

In this way, it becomes necessary to know the competences that nurses must acquire so that their professional performance is based on the principles of prevention and control of HCRI. Enabling these competencies to be developed in the training process during the undergraduate course represents a great challenge.

Conceptually, competence is the articulation of three dimensions: knowledge, skills and attitudes necessary to reach a given objective^(^ ^10^ ^)^. In the year 2015, a systematized research was carried out in the literature to identify studies that would serve as foundations for the generalist nurse training process. However, all the works found were international, from the United States, Canada, the United Kingdom, Australia and Taiwan^(^ ^11^ ^-^ ^14^ ^)^, which describe competencies for generalist nurses and specialists in prevention and control of HCRI.

Many aspects of HCRI prevention and control, such as evidence-based prevention indicators, apply in any country. However, considering the particularities that permeate the professional practice, influenced by the organization of the health systems of each country and in view of the identification of this gap in the Brazilian nursing production, the present study aimed to determine the competences for the prevention and control of HCRI that must be developed by the nurse in Brazil, constituting a guiding axis to rethink the teaching of this theme.

In addition, the objective is to differentiate the skills of the generalist and specialist nurse in this area, contributing to the advancement of debates, teaching and practices of nurses, becoming a support for other countries to use this classification to validate and develop skills of nurses in their territories.

This research aims to define the competencies for the prevention and control of healthcare-related infections that should be developed by the generalist nurse and by the specialist nurse in infection control in Brazil.

## Method

The present study was developed using the Delphi technique, an efficient method to generate consensus on a complex problem, based on the opinion of experts in the subject^(^ ^15^ ^-^ ^16^ ^)^.

Participants were Brazilian graduated professionals with expertise in the area of infection prevention and control. To define the participants, two steps were taken. The first consisted of analyzing a list of possible lecturers of the Brazilian Congress of Infection Control and Hospital Epidemiology in the years 2010, 2012 and 2014; researchers from the research groups registered in the Directory of Research Groups in Brazil that were working on the topic; members of the board of directors of the Brazilian Association of Professionals on Infection Control and Hospital Epidemiology of 2011/2012, 2013/2014, 2015/2016; and teachers of postgraduate courses in the area.

Subsequently, the professionals’ curricula were consulted by applying the criteria: being a nurse, physician or pharmacist; having published an article on the subject in the last 10 years; having published a summary on the subject in a national or international event in the last 10 years; being a lecturer in postgraduate course in the area for more than 5 years; and having more than 10 years of professional experience in committees or infection control services.

After the selection of the participants, a total of 175 professionals were invited to participate by means of an electronic message. Of these, 39 (31 nurses and 8 physicians) accepted it and signed the Informed Consent Form.

There were representatives from the South, Southeast, Midwest, Northeast regions; none from the North region responded to the contacts. The participants had an average of 26 years of graduation, 62% had a doctorate, 36%, master’s degree and 2%, specialization. Regarding the professional experience, the relation of the length of time working in the area with the average in years was in health services 92%/17 years, infection control committees and services 74%/13 years, and undergraduate education in the health area 95%/5 years.

**Figure 1:**
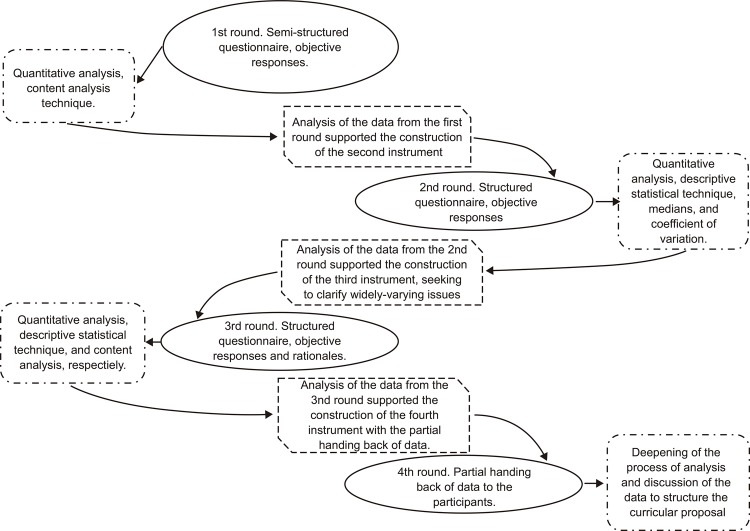
– Conduction of the Delphi technique. Florianópolis, Santa Catarina, Brazil, 2017

Source: ^(^^16^^)^.

Four rounds were conducted in the period between August 2015 and March 2016, according to Figure 1. In the first, participants were asked to indicate at least three competencies for the prevention and control of infections for the generalist nurse and three others for the nurse specialist in this area.

At the end of the round, with a return of 39 participants, a list of 143 competencies was generated for the general nurse and 150 for the specialist nurse. These data were organized and analyzed according to the principles of content analysis^(^ ^17^ ^)^, considering the classification of core, generic and specific competences.

Core competencies correspond to those common to all health professions. The generic ones are those common to a professional field of knowledge and, in this study, are considered the competences for the infection control that the generalist nurse must have developed upon graduation.

Specific competencies correspond to those inherent in a profession or specialty. In this study, it encompasses the competencies expected for a specialist nurse to perform the infection control and that they need to acquire at the end of the specialization course in the area of prevention and control of HCRI.

After the analysis of this round, a list of competences was elaborated, which consisted of 10 core competencies, 15 generic competences and 17 specific competences. These competences composed the instrument of the second round, so that the participants evaluated them according to the degree of importance assigned to each one using a Likert scale (0-none, 1-very little, 2-little, 3-fair, 4- great and 5-very great).

With the answers of the second round, which counted with the participation of 35 experts, the statistical analysis was carried out to define the degree of agreement of each competence. The items that reached the median 5 and presented coefficient of variation below 20% were considered as consensus by the group.

The competencies that had not reached consensus in this round composed the instrument of the third round, with the participation of 30 experts. The data were analyzed through descriptive statistics (median and coefficient of variation) and composed the instrument of the fourth round, which contained the return of the results.

This work followed the precepts of Resolution 466/2012 and was approved by the Ethics Committee in Research with Human Beings, number 36739714.4.0000.5355.

## Results

The results are presented from a list with the competences (name and description) that obtained a consensus among the participants, considering their classification, ending with 4 core, 14 generic and 17 specific competences, according to Tables 1, 2 and 3, respectively.

**Table 1:** Core competencies for the nurse in the prevention and control of healthcare-related infections. Florianópolis, Santa Catarina, Brazil, 2015 – 2016

Core Competencies	Coefficient of variation*
Education and professional development	11%
Communication	10%
Decision making	10%
Ethics	9%

*It was considered as consensus the competences that reached median 5 and coefficient of variation less than 20%

**Table 2:** Generic competencies for nurses in the prevention and control of healthcare-related infections. Florianópolis, Santa Catarina, Brazil, 2015 – 2016

Generic competencies	Coefficient of variation*
Maintenance of aseptic chain	20%
Health services waste management	18%
Recognition of the problem of healthcare-related infections	17%
Collaboration with epidemiological surveillance and knowledge of the epidemiological profile of health services	17%
Management of exposure to biological material	17%
Implementing infection prevention actions	17%
Cleaning, disinfection and sterilization of health products/equipment	13%
Cleaning and disinfection of environments and surfaces	12%
Care for the infected patient	12%
Use of standard and specific precautions	11%
Recognition of the process of the microbial transmission chain	11%
Identification of risk for infection	11%
Use of Personal Protective Equipment	9%
Hand hygiene	7%

*It was considered as consensus the competences that reached median 5 and coefficient of variation less than 20%

**Table 3:** Specific competences for the specialist nurse in the prevention and control of healthcare-related infections. Florianópolis, Santa Catarina, Brazil, 2015 – 2016

Specific competences	Coefficient of variation*
Maintenance of aseptic chain^†^	18%
Management of the Committee and Service of Control of Healthcare-Related Infections	15%
Monitoring of use of antimicrobials	15%
Evaluation of inputs and materials	12%
Monitoring the development of multiresistant microorganisms	12%
Health services waste management^†^	10%
Interaction with the various sectors of the health service	9%
Indication /Maintenance of invasive devices and procedures	8%
Cleaning and disinfection of environments and surfaces^†^	7%
Cleaning, disinfection and sterilization of health products^†^	7%
Performing epidemiological surveillance	7%
Performing Process and structure surveillance	7%
Developing and implementing manuals, standards and protocols for the prevention and control of healthcare-related infections	6%
Elaborating and implementing the Program for the Control of Healthcare-Related Infections	6%
Hand hygiene^†^	6%
Use of standard and specific precautions^†^	6%
Use of personal protective equipment^†^	6%

*It was considered as consensus the competences that reached median 5 and coefficient of variation less than 20%;†The dimensions mentioned in the generic competences are added here.

Among the core competencies, the *education and professional development* consists of seeking new knowledge to improve their practice, knowing the various teaching strategies to use with health professionals, patients and companions, developing and participating in HCRI prevention and control training programs and working together with their team with an observant and proactive attitude, thus becoming a multiplier agent of knowledge and skills.

*Decision making* consists of assessing the needs of the service and the patient, organizing and systematizing the information from the scientific evidence and available resources, and *communication* is characterized by knowing the forms of communication, ensuring that communication is understandable, effective and favors teamwork and health care safety.

*Ethics* means to understand the concept of ethics and work in an ethical way in all the situations in which one is involved.

The generic competences for the control of infections for the generalist nurse make up Table 2.

*Maintenance of the aseptic chain* is understood as the competence to recognize the process of the chain of transmission, to know the principles of asepsis and antisepsis, to master the techniques necessary for the maintenance of asepsis of a procedure, recognize and intervening when there is a breakdown of asepsis during a procedure.

*Health services* w*aste management,* which is a routine in all units of health institutions, consists of knowing the definition and classification of the term, and the care in separation and management, especially with infectious and sharp objects. It also includes supervising waste disposal in one’s sector.

*Recognition of the problem of HCRI* means to identify the problem of HCRI and its implication for healthcare, working proactively, collaborating with the sector responsible for the control of HCRI.

*Collaborating with epidemiological surveillance and knowing the epidemiological profile of health services* consists of perceiving the signs and symptoms of infections, identifying patients with signs and symptoms of infections, recognizing the epidemiological surveillance system, monitoring epidemiological indicators, discussing HCRI cases and implementing actions to prevent and control infections.

*Management of exposure to biological material* includes knowing the preventive measures before and after accidents with exposure to biological material and institutional flow and knowing which vaccines are recommended for health professionals.

*Implementing infection prevention actions* consists in knowing the measures for the prevention of infections, identifying the risks and establishing the preventive measures, identifying and implementing specific measures to prevent infections associated with invasive devices and procedures, recognizing and early identifying the signs and infectious symptoms.

The competence *Cleaning, disinfection and sterilization of health products/equipment* includes differentiating cleaning, disinfection and sterilization, the classification according to potential contamination (critical, semi-critical, non-critical), forms of contamination and dissemination of microorganisms, procedures, products and materials used in processing.

*Cleaning and disinfection of environments and surfaces* means to know the classification of environments and surfaces according to potential contamination, forms of contamination and dissemination of microorganisms and supervise the performance of these procedures in one’s sector.

*Care for the infected patient* consists of knowing the pathophysiology of the infections and the appropriate therapy.

*Use of standard and specific precautions* includes recognizing the forms and routes of transmission of microorganisms, knowing the types and indications of standard and specific precautions, mastering the techniques of attire, and identifying patient with multi-resistant microorganisms and the care in the management thereof.

*In order to recognize the process of the microbial transmission chain,* it is necessary to know the intrinsic and extrinsic forms and factors of transmission and dissemination of microorganisms, differentiate colonization, contamination and infection, and reservoir, vector and host and identify risk situations and work by breaking the microbial transmission chain.

*Identifying risks for infection* requires knowing the chain of transmission of microorganisms, identifying and intervening early in face of risks for infection development, encouraging and supervising the team regarding standards and recommendations for reducing the risk for infection spread.

*Use of Personal Protective Equipment (PPE)* requires knowledge of the types of PPE, indication of use, indication of disposal, substitution or reprocessing of each item, mastering equipment placement and removal techniques, supervising the team regarding the use of PPE and participating in the standardization and testing of PPE.

*Hand hygiene (HH)* involves the knowledge about microorganisms of the hands, contaminating microorganisms, understanding the importance of HH, mastering the techniques for carrying out HH and the necessary materials, encouraging the team to perform HH and evaluating the indicators of adherence to the procedure.

Specific competencies for infection control for the specialist nurse in infection prevention and control include core and generic skills.

*Maintenance of aseptic chain* encompasses conducting interventions in the event of an aseptic break in a procedure and monitoring the case.

*Management of the Committee and Service of Control of Healthcare-Related Infections* consists of knowing historical aspects and legislation related to the committee and the control service of HCRI, knowing the epidemiological situation of HCRI globally, developing management reports on HCRI in the service, managing institutional discussions on HCRI, its prevention and control and disclosing the theme in the service.

*Monitoring of use of antimicrobials* consists of knowing the mechanisms of action of antimicrobials, the relationship between sensitivity and resistance to antimicrobials, monitoring the results of antibiograms, disclosing the institutional protocol for the therapeutic and prophylactic use of antimicrobials and monitoring the rational use of antimicrobials.

*Evaluation of inputs and materials* consists of evaluating and standardizing inputs and materials in the service related to HCRI, as well as analyzing the cost-benefit ratio.

*Monitoring the development of multiresistant microorganisms* consists of knowing the mechanisms of resistance of microorganisms, monitoring the development of multiresistant microorganisms in the national and international scope, monitoring the cultures and the resistance level of the microorganisms in the service, identifying patients with multiresistant microorganisms and elaborating protocols for the management of these patients.

*Health services waste management* involves participating in the elaboration of the Waste Management Plan of the Health Service, raising awareness and training professionals to correctly manage health service waste.

*Interaction with the various sectors of the health service* means to be a reference in the service for doubts and solution of problems related to HCRI, encourage the health team in adhering to the practices of prevention and control of infections and participate in the elaboration of procedures and routines.

*Indication/Maintenance of invasive devices and procedures* consists of developing indicators on use, length of stay and infectious complications arising from invasive devices and procedures, creating strategies for improving adherence of health professionals to best referral and maintenance practices.

*Cleaning and disinfection of environments and surfaces* encompasses working in partnership with the cleaning service, elaborating protocols and indicators for cleaning and disinfection of environments and surfaces.

*Cleaning, disinfection and sterilization of health products* encompasses knowing the laws, approving protocols for cleaning, disinfection, sterilization and single use item processing policy; developing and analyzing indicators for the evaluation of processing.

*Performing epidemiological surveillance* consists of mastering the principles of statistics and epidemiology, developing an epidemiological surveillance system, collecting, analyzing and disseminating infection rates, recognizing major infections and the profile of micro-organisms, investigating the cause of infections, monitoring and managing epidemiological indicators, investigating and manage infectious outbreaks and issuing periodic reports on the situation of the HCRI in the health service.

*Performing process and structure surveillance* means to carry out periodic monitoring of processes and structures related to HCRI prevention, set short, medium and long term goals for health care improvement, generate indicators to evaluate and monitor the processes and structures.

*Developing and implementing manuals, standards and protocols for the prevention and control of HCRI* means to accompany the scientific evidence related to the prevention and control of HCRI, elaborate, disclose and implement policies, manuals, norms and protocols of assistance and identify risks for infections.

*Elaborating and implementing the Program for the Control of HCRI* consists of developing and implementing the program based on legislation, assessing the effectiveness of the program, reshaping actions according to results and having knowledge about management and continuous quality improvement programs.

*Hand hygiene* involves investigating the reasons for non-adherence to HH, developing strategies to encourage HH, creating and evaluating indicators of adherence to the procedure.

*Use of standard and specific precautions* encompasses developing protocols and indicators of adherence to specific precautions and develop visual communication system for specific precautions.

The competence *use of personal protective equipment* encompasses encouraging the appropriate use of PPE among health professionals and working collaboratively with the job security service.

The organization of these competences allows an understanding between the differences of performance of the generalist nurse and the specialist nurse in infection control. The general practitioner is important in the process of infection prevention and control in the consolidation of daily activities in health services.

## Discussion

From the definition of competences for the prevention and control of HCRI, it was verified that the core competences - communication, ethics, decision-making, education and professional development - are in line with the findings of national^(^ ^18^ ^-^ ^20^ ^)^ and international^(^ ^21^ ^)^ studies that emphasize the importance of these competencies for health professionals. These are structural elements in the process of production of health care, favoring the safety of patients and professionals in health services, as well as their quality and efficiency.

The generalist nurse is considered to be a professional capable of developing care at any point in the health care network and, therefore, their training must encompass knowledge, skills and attitudes that enable the student to develop competences for the comprehensive care of the clients of the different health services^(^ ^22^ ^)^. Thus, the generic competences for the prevention and control of HCRI are crucial in the training of the generalist nurse, since the issue of HCRI is present in all services.

The dissemination of microorganisms with high potential for pathogenicity and microbial resistance in specialized health care environments and community infections are also examples of the contribution of all health professionals to the prevention and control of HCRI for the health of the community.

The generic competences for the generalist nurse in the prevention and control of HCRI listed and that obtained consensus among the Brazilian experts are in line with the competences that were presented in other international studies that approached this subject^(^ ^11^ ^,^ ^13^ ^-^ ^14^ ^)^. This fact evidences that the problem of HCRI is worldwide and, although there are epidemiological differences between regions, prevention and control measures are applicable in any context and, therefore, are necessary for the training of nurses in a similar way.

A pioneer study^(^ ^14^ ^)^ in the definition of competencies for the prevention and control of infections for health professionals, conducted Canada in the year of 2006, has been used as a parameter by the subsequent studies. An American study^(^ ^13^ ^)^ also adds competence to the management of disasters involving infectious diseases.

Thus, it becomes evident the importance of a national study that contemplates the vision of Brazilian professionals with expertise in HCRI prevention and control, favoring the delineation of competences according to the needs of the national health services.

Regarding the specific competences that the specialist nurse in infection control needs to have built at the end of his/her specialization, a great consonance of these findings was identified with the competencies that are presented in international studies^(^ ^12^ ^,^ ^23^ ^-^ ^24^ ^)^.

It is important to point out the importance of the specialist nurse, who is responsible for program management and for articulating government recommendations and research that produces guidelines and evidence to support HCRI prevention and control actions.

The definition of competencies for the prevention and control of HCRI for generalist and specialist nurses is not intended to evidence or promote hyperspecialization, but rather to emphasize the importance of general practitioners so that the consolidation of these practices contributes effectively to the quality and safety of health care.

As HCRI prevention and control are a recurring theme in all health care areas and have a differentiated connotation at the time of its approach and development during undergraduation, they must be developed so that the student is prepared for the application of these generic skills for the prevention and control of HCRI in all health services.

The differentiation of generic and specific competences does not intend to fragment or isolate them, but to distinguish them, differentiating them and at the same time situating them in the environment in which they are inserted, exist and interact with the various elements of health care. The knowledge of these competences allows the comprehension of the generality that is included in the specialty of this subject, favoring its visualization and understanding through the functions and attributions of each nurse.

Future studies can contribute to the improvement of the description of these competences, detailing them from the dimensions of a competence: knowledge, skills and attitudes^(^ ^10^ ^)^, thus contributing to the expansion of the pathways for its implementation in nursing undergraduate courses and specialization courses.

The absence of participants from the Northern region of Brazil constitutes a limitation of this study. However, considering the peculiar universality of the identified competencies and, still, the similarity found with international studies, we believe that the results can be extended to that region. Still, we believe that it can contribute to researchers and professionals from other countries that seek to develop the subject in their territory, being the basis for the organization of these competences.

## Conclusion

From Brazilian professionals with expertise in the area of prevention and control of HCRI, it was possible to develop and register generic and specific competences for the national scenario.

The definition of competencies for the prevention and control of HCRI is the first step to begin the rethinking of the teaching and learning process in the initial training of nurses, establishing the moments in which each competency is developed throughout the course.

Thus, the competencies defined in this study can contribute to the expansion of the discussions about the teaching process in nursing undergraduate courses and generate subsidies for the creation of instruments to evaluate its consolidation among nursing students. Similarly, among nursing professionals, these competencies can be used to define permanent health education programs.

In addition, these results can contribute in the international scope, since there is a global need of restructuring of the teaching of these competences during the training of the new nurses.

## References

[1] Giroti SKO, Garanhani ML (2015). Infections related to health care in nurses’
education. Rev Rene.

[2] Giroti SKO, Garanhani ML, Guariente MHDM, Cruz EDA (2013). Teaching of Health Care-Related Infections within an Integrated
Nursing Curriculum. Creative Education.

[3] Valle AR, Andrade D, Sousa AF, Carvalho PR (2016). Infection prevention and control in households: nursing
challenges and implications. Acta Paul Enferm.

[4] Sousa AFL, Matos MCB, Matos JGNF, Sousa LRM, Moura MEB, Andrade D (2017). Prevention and control of infection in professional 199 nursing
training: a descriptive study. Online braz j nurs.

[5] Darawad MW, Al-Hussami M (2013). Jordanian nursing students’ knowledge of, attitudes towards, and
compliance with infection control precautions. Nurse Educ Today.

[6] Halboub ES, Al-Maweri SA, Al-Jamaei AA, Tarakji B, Al-Soneidar WA (2015). Knowledge, attitudes, and practice of infection control among
dental students at Sana’a University, Yemen. J Int Oral Health.

[7] Xiong P, Zhang J, Wang X, Wu TL, Hall BJ (2017). Effects of a mixed media education intervention program on
increasing knowledge, attitude, and compliance with standard precautions
among nursing students: A randomized controlled trial. Am J Infect Control.

[8] Hinkin J, Cutter J (2014). How do university education and clinical experience influence
pre-registration nursing students’ infection control practice? A
descriptive, cross sectional survey. Nurse Educ Today.

[9] Ward DJ (2013). The barriers and motivators to learning infection control in
clinical placements: interviews with midwifery students. Nurse Educ Today.

[10] Almeida ML, Peres AM (2012). Knowledge, skills, and attitudes towards management of nursing
graduates of a Brazilian public university. Invest Educ Enferm.

[11] Liu LM, Curtis J, Crookes PA (2014). Identifying essential infection control competencies for newly
graduated nurses: a three-phase study in Australia and
Taiwan. J Hosp Infect.

[12] European Centre for Disease Prevention and Control (2013). Core competencies for infection control and hospital hygiene
professionals in the European Union.

[13] Carrico RM, Rebman T, English JF, Mackey J, Cronin SN (2008). Infection prevention and control competencies for hospital-based
health care personnel. Am J Infect Control.

[14] Henderson E (2016). Infection prevention and control core competencies for health
care workers: a consensus document. Can J Infect Control.

[15] Marques JBV, Freitas D (2018). The delphi method: characterization and potentialities for
educational research. Pro-Posições.

[16] Massaroli A, Martini JG, Lino MM, Spenassato D, Massaroli R (2017). The delphi method as a methodological framework for research in
nursing. Texto Contexto Enferm.

[17] Silva AH, Fossá MIT (2015). Content analysis: example of application of the technique for
analysis of qualitative data. Qual Rev Elet.

[18] Moyano LG (2015). The ethics of caring and its application in nursing
profession. Acta Bioethica.

[19] Busanello J, Lunardi WD, Kerber NPC (2013). Nurses’ subjectivity production and the decision-making in the
process of care. Rev Gaúcha Enferm.

[20] Backes VMS, Prado ML, Lino MM, Ferraz F, Reibnitz KS, Canever BP (2012). Nursing Education Research Groups in Brazil. Rev Esc Enferm USP.

[21] Expósito JS, Costa CL, Agea JLD, Izquierdo MDC, Rodríguez DJ (2018). Ensuring relational competency in critical care: importance of
nursing students communication skills. Intensive Crit Care Nurs.

[22] Turrini RNT, Costa ALS, Peniche ACG, Bianchi ERF, Cianciarullo TI (2012). Education in operating room nursing: transformation of the
discipline at University of São Paulo School of Nursing
(Brazil). Rev Esc Enferm USP.

[23] Kim KM, Choi JS (2015). Self-perceived competency of infection control nurses based on
Benner’s framework: a nationwide survey in Korea. App Nurs Res.

[24] Murphy DM, Hanchett M, Olmsted N, Farber MR, Lee TB, Haas JP (2012). Competency in infection prevention: a conceptual approach to
guide current and future practice. Am J Infect Control.

